# Gas‐Phase Integration of Trophically Distinct Microbial Cultures for Net‐Reduced CO_2_ and Enhanced Metabolite Production

**DOI:** 10.1111/1751-7915.70282

**Published:** 2025-12-20

**Authors:** Jaeyoung Yu, Danbee Kim, Jiye Lee, Hui Su Kim, Hwi Jong Jung, Yuri Kim, Sahng Hyuck Woo, Eunsung Kan, Jeong Hyeon Kim, Soo Youn Lee

**Affiliations:** ^1^ Department of Biotechnology and Life Science Tokyo University of Agriculture and Technology Tokyo Japan; ^2^ Gwangju Clean Energy Research Center Korea Institute of Energy Research Gwangju Republic of Korea; ^3^ Department of Environmental Health and Engineering, Whiting School of Engineering Johns Hopkins University Baltimore Maryland USA; ^4^ Department of Chemical Engineering Chonnam National University Gwangju Republic of Korea; ^5^ Department of Biotechnology and Bioengineering Chonnam National University Gwangju Republic of Korea; ^6^ Jeju Global Research Center Korea Institute of Energy Research Jeju Republic of Korea; ^7^ Department of Biological and Agricultural Engineering & Texas A&M AgriLife Research Center Texas A&M University Stephenville Texas USA; ^8^ Public CMO for Microbial‐Based Vaccine Hwasun‐gun Jeollanam‐do Republic of Korea

**Keywords:** adaptive laboratory evolution, circular biorefinery, CO_2_ reassimilation, gas‐linked co‐culture system, *Rhodobacter sphaeroides*

## Abstract

The continued increase in atmospheric CO_2_ concentrations has intensified global efforts to develop sustainable biotechnologies that capture and reutilise carbon rather than releasing it. While photosynthetic microorganisms provide a renewable route for CO_2_ fixation into organic products, heterotrophic fermentation remains the industrial standard due to its high productivity, controllability and scalability. Consequently, integrating the carbon efficiency of autotrophic processes with the productivity of heterotrophic systems may represent a promising strategy toward circular biomanufacturing. Here, we developed a gas‐linked co‐culture system designed to spatially separate heterotrophic and autotrophic metabolisms while enabling gas‐phase CO_2_ exchange between them. This configuration allowed CO_2_ released during heterotrophic metabolism to be reutilised in autotrophic metabolism, supporting cooperative carbon cycling. Compared to non‐linked controls, the gas‐linked system enhanced biomass accumulation and nearly doubled the production of value‐added metabolites—namely polyhydroxybutyrate (PHB) and carotenoids—while reducing net CO_2_ emissions by 20.62%. Although further optimisation is necessary to approach a fully net‐zero process, this study demonstrates that gas‐phase integration of trophically distinct cultures offers a promising platform for circular carbon biorefineries.

## Graphical abstract

A gas‐linked co‐culture system that enables spatially separated yet metabolically connected microbial interactions has been found to enhance the sustainable production of value‐added metabolites while eliminating CO_2_ emissions.

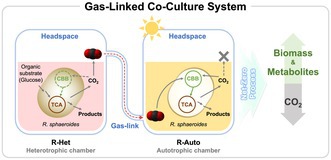

## 1 Introduction

The continued rise in atmospheric CO_2_ concentrations has intensified global efforts to develop sustainable biotechnologies that capture and reutilise carbon rather than emitting it. A central concept in this context is the circular carbon economy, which aims to close the carbon loop by continuously recycling carbon within industrial systems (Alsarhan et al. 2021; Favoino et al. 2025). Among various strategies for carbon recycling, biological CO_2_ fixation via photosynthesis offers a direct and renewable route to convert inorganic carbon into organic biomass and value‐added chemicals (Almomani et al. 2023). For instance, photosynthetic microorganisms such as microalgae, cyanobacteria, and purple non‐sulfur bacteria not only fix CO_2_ through the Calvin‐Benson‐Bassham (CBB) cycle catalysed by RuBisCO but also produce a wide range of metabolites. Many species, in particular, accumulate energy‐dense lipids or polyhydroxybutyrate (PHB) as storage forms of fixed carbon (Lee et al. 2022). Through this capability, photosynthetic microbes represent attractive platforms for bio‐based production of fuels and materials, offering a sustainable alternative to fossil‐based processes. These efforts align with several of the United Nations Sustainable Development Goals (SDGs), notably SDG 12 (Responsible Consumption and Production) and SDG 13 (Climate Action) (Sachs et al. 2022).

However, conventional heterotrophic fermentation remains the dominant approach in industrial biotechnology. These processes depend on reduced carbon substrates, such as sugars or other feedstocks, and inevitably release CO_2_ as a metabolic byproduct (Kurt et al. 2023). In traditional fermentations, more than one‐third of the input sugar carbon is typically lost as CO_2_ (Jones et al. 2016), leading not only to increased greenhouse gas emissions but also to substantial carbon loss from the feedstock. Despite these environmental concerns, heterotrophic fermentation continues to serve as a central platform for large‐scale biochemical production owing to its high productivity, robust process control, and compatibility with established industrial infrastructure (Augustin et al. 2024; Nielsen et al. 2022). Consequently, improving the carbon efficiency of these conventional systems is essential for achieving both economically viable and environmentally sustainable biomanufacturing.

One effective approach to improve carbon efficiency is to capture and reutilise CO_2_ emitted from heterotrophic processes, thereby reducing carbon loss. In this regard, the integration of heterotrophic and autotrophic cultivation presents a strategic opportunity to couple the productivity of heterotrophs with the carbon fixation capacity of autotrophs, a strategy that enables more circular and sustainable bioprocesses (Ronan et al. 2021). Synthetic microbial consortia—typically combining heterotrophic bacteria with photoautotrophs (i.e., microalgae or cyanobacteria)—have been explored to facilitate mutual metabolic exchange. These systems show the potential to enhance total carbon conversion efficiency through metabolic cross‐feeding. Nevertheless, the co‐cultivation of physiologically distinct species introduces substantial technical challenges, including mismatched growth rates, differences in nutrient and light requirements, and competition for shared resources (Zuñiga et al. 2020). In some cases, excess organic carbon can suppress photoautotrophic CO_2_ fixation, while nutrient competition may destabilise population dynamics. Although strategies such as barrier‐separation (e.g., using membranes or agar to inhibit direct contact while permitting diffusional exchange) or metabolic engineering have been attempted to improve co‐culture stability (Bohutskyi et al. 2024), the industrial application of mixed species systems remains limited.

To address these limitations, we established a gas‐linked co‐culture system designed to integrate heterotrophic and autotrophic metabolism while preventing direct interspecies interference. In this system, the two cultures are physically separated yet connected through gas‐linked chambers that allow CO_2_ transfer, thereby maintaining metabolic exchange without nutrient competition or cell‐to‐cell contact. In this study, we evaluated whether this configuration could support metabolic cooperation in which CO_2_ released during heterotrophic metabolism is reutilised in autotrophic metabolism by comparing its performance with that of a non‐linked control. For proof of concept, *Rhodobacter sphaeroides* was employed as a model species, and two functionally specialised populations of the same organism—adapted to heterotrophic and autotrophic growth, respectively—were derived via adaptive laboratory evolution (ALE). We propose that this gas‐linked co‐culture system holds promise as a modular framework for closing the carbon loop in microbial production systems and advancing sustainable biomanufacturing.

## 2 Experimental Procedures

### 2.1 Bacterial Strain and Baseline Cultivation

#### 2.1.1 Bacterial Strain

*Rhodobacter sphaeroides* KCTC 1434 was obtained from the Korean Collection for Type Cultures (KCTC). The strain was cryopreserved as a 50% (v/v) glycerol stock and stored at −80°C until use.

#### 2.1.2 Baseline Cultivation

To assess the fundamental metabolic responses of non‐adapted *R. sphaeroides* to varying carbon conditions, a baseline culture was established prior to trophic adaptation. Cryopreserved cells were thawed and inoculated at 10% (v/v) into 100 mL of 1× Sistrom medium (Sistrom 1962) supplemented with different glucose concentrations (0, 1, 3, or 5 mM) in sterile 250 mL serum bottles sealed with butyl rubber stoppers and aluminium crimp caps (hereafter referred to as sealed serum bottles). Two different headspace gas conditions were tested: (i) a gas mixture of 60% H_2_, 5% CO_2_ and 35% Ar and (ii) a 100% Ar atmosphere. Argon was selected instead of nitrogen to avoid physiological effects associated with N_2_ fixation and was therefore consistently used as the inert purge gas throughout this study. All cultures were incubated at 30°C with agitation at 150 rpm under continuous light irradiation (400–600 lx), which was defined as the standard condition. Growth, headspace CO_2_ concentration, and residual glucose were monitored over time.

### 2.2 Adaptive Cultivation of *R. sphaeroides*

#### 2.2.1 Heterotrophic Adaptation of *R. sphaeroides* (R‐Het)

Cells were inoculated at 10% (v/v) into 150 mL of 1× Sistrom medium containing succinic acid as the organic carbon source. Cultivation was performed in 250 mL sterile Erlenmeyer flasks sealed with silicone stoppers aerobically under standard conditions for 2 days. After incubation, cells were harvested and washed twice with carbon‐free 1× Sistrom medium by centrifugation (4200 rpm, 28°C, 15 min), then resuspended in fresh Sistrom medium containing succinic acid to an optical density at 660 nm (OD_660_) of approximately 0.1. A 150 mL aliquot of this suspension was then transferred into fresh sterile Erlenmeyer flasks and incubated under the same conditions. This adaptation cycle (harvesting, washing, OD adjustment, cultivation) was repeated three times to promote heterotrophic growth.

#### 2.2.2 Autotrophic Adaptation of *R. sphaeroides* (R‐Auto)

For autotrophic adaptation, cells were initially pre‐cultured heterotrophically to build biomass, then harvested and washed as described above. The pellet was resuspended to an OD_660_ of approximately 0.1 in carbon‐free Sistrom medium and transferred (150 mL) into sterile 250 mL sealed serum bottles. The headspace was replaced with 60% H_2_, 10% CO_2_ and 30% Ar, after which cultures were incubated under standard conditions for 14 days. After 14 days of incubation, cells were harvested, washed and reinoculated into fresh carbon‐free medium. This cycle was repeated three times to ensure stable autotrophic growth.

### 2.3 Evaluation of Adapted Cells Under Glucose‐Supplemented Conditions

To assess CO_2_ dynamics, heterotrophically (R‐Het) and autotrophically (R‐Auto) adapted cells were cultivated under glucose‐supplemented conditions. After harvesting and washing, cultures were resuspended to OD_660_ = 0.1 in 1× Sistrom medium containing 1 mM glucose. A 20 mL aliquot was then transferred into a sterile 120 mL sealed serum bottle, and the headspace was purged with 100% Ar. Cultures were incubated under standard conditions for 14 days. For R‐Auto cultures, an additional 1 mM glucose was added after the initial 14‐day period to examine the persistence of CO_2_ reassimilation. Growth, headspace CO_2_ concentration, and residual glucose were monitored.

### 2.4 Gas‐Linked Co‐Culture System

To spatially separate CO_2_‐generating and CO_2_‐assimilating processes, a gas‐linked co‐culture system was established. Two culture chambers were prepared: one containing R‐Het cells and the other containing R‐Auto cells. R‐Het and R‐Auto were harvested, washed with carbon‐free medium, and resuspended to OD_660_ = 0.1 in 1× Sistrom medium containing 5 mM glucose and carbon‐free medium, respectively. Then, 200 mL of each suspension was transferred into sterile 250 mL GL45 glass bottles equipped with multiple ports for sampling and gas exchange. Chambers were sealed with a screw cap, and headspaces were purged with 100% Ar.

In the non‐linked control, chambers operated independently without any gas exchange. In the gas‐linked system, headspace gas from the R‐Het chamber was circulated into the liquid phase of the R‐Auto chamber using a sterile peristaltic pump (0.1 vvm), and headspace pressures were equalised via an additional gas line. Cultures were incubated at 28°C and 150 rpm under continuous light (200–400 lx) for 12 days. Growth, CO_2_ concentration, and glucose were monitored throughout. Intracellular metabolites (carotenoids and PHB) were analysed at the end of cultivation.

### 2.5 Analytical Methods for Physiological and Metabolic Parameters

#### 2.5.1 Microbial Growth

OD_660_ was measured using a UV/Vis spectrophotometer (Eppendorf BioSpectrometer, Eppendorf AG, Germany) with disposable UVette cuvettes. Culture samples were aseptically collected using sterile syringes either through the rubber stoppers of serum bottles or via the sampling ports of GL45 bottles. Each measurement was performed using 50 μL of culture.

#### 2.5.2 Headspace CO_2_ Concentration

Headspace CO_2_ concentrations were determined by gas chromatography (GC) according to a previously described method (Kim et al. 2024). Gas samples (100 μL) were collected using a sterile gas‐tight syringe and analysed on an Agilent 7890 GC System (Agilent Technologies, USA) equipped with a molecular sieve/Porapak N column and a thermal conductivity detector. The GC oven temperature was maintained at 40°C, with injector and detector temperatures set to 150°C and 200°C, respectively. Helium was used as the carrier gas at a flow rate of 2 mL/min. CO_2_ concentrations were generally expressed in mM; in specific cases requiring μmol, values were calculated by multiplying the measured concentration by the headspace volume.

#### 2.5.3 Glucose Concentration

Residual glucose concentrations were measured by high‐performance liquid chromatography (HPLC) following the procedure of Lee, Fitriana, et al. (2020). After sample collection (50 μL), cells were removed by centrifugation, and the supernatant was filtered using a 0.2 μm pore‐size syringe filter. The filtrate was analysed using an Agilent 1260 Infinity HPLC system (Agilent Technologies) with a UV detector set at 210 nm. Glucose separation was achieved using an Aminex HPX‐87H column (300 mm × 7.8 mm i.d.; Bio‐Rad Laboratories, USA) under isocratic elution with 5 mM sulfuric acid at 0.6 mL/min. The column was maintained at 50°C, and the injection volume was 20 μL. Calibration curves were generated using standard glucose solutions.

#### 2.5.4 Quantification of Intracellular PHB

Quantification of intracellular PHB was performed using a modified spectrophotometric assay (Li et al. 2023). After cultivation, 10 mL of culture was transferred to polypropylene tubes that had been pre‐cleaned with ethanol and hot chloroform to eliminate residual plasticisers. Transferred samples were centrifuged at 4200 rpm and 4°C for 15 min. Cell pellets were resuspended in a 5% sodium hypochlorite solution and incubated at 37°C for 30 min to facilitate cell lysis. The lysate was centrifuged, and the pellet was sequentially washed with acetone and ethanol. PHB granules were then extracted by dissolving the pellet in 1 mL of chloroform and incubating at room temperature (RT) for 12 h. A 100 μL aliquot of the extract was dried at 50°C to remove chloroform. Concentrated sulfuric acid (10 mL) was then added to convert PHB to crotonic acid by heating at 100°C for 20 min in a water bath. After cooling to RT, the solution was mixed thoroughly, and absorbance was measured at 235 nm using a UV/Vis spectrophotometer (Eppendorf BioSpectrometer). Sulfuric acid and PHB standards (Catalogue No. 363502, Sigma‐Aldrich, USA), treated identically, were used as a blank and for calibration, respectively. PHB content was reported as total production or cell‐specific yield normalised to dry cell weight (DCW). DCW was measured from freeze‐dried 10 mL culture aliquots.

#### 2.5.5 Quantification of Intracellular Carotenoids

Carotenoid content was determined spectrophotometrically according to Lee, Fitriana, et al. (2020). After cultivation, 100 mL of culture was freeze‐dried. A 10 mg portion of dried biomass was treated with 1 mL of 3 M HCl at 30°C with shaking (100 rpm) for 30 min to disrupt the cell wall structure and release intracellular pigments. After centrifugation at 10,000 rpm for 20 min, the pellet was resuspended in 1 mL of acetone and incubated under identical conditions (30°C, 100 rpm, 30 min) to extract carotenoids into the solvent phase. The extract was centrifuged, and the supernatant was used for measurement. Absorbance at 480 nm was recorded using a UV/Vis spectrophotometer (Eppendorf BioSpectrometer), and carotenoid concentrations were calculated using a conversion factor of 0.16 μg/mL per A_480_. Results were expressed as total production or cell‐specific yield.

### 2.6 Statistical Analysis

All experiments were independently performed at least twice. Data are expressed as mean ± standard deviation. Statistical significance between groups was determined using Student's *t*‐test, with a significance threshold of *p* < 0.05. Graphs and data plots were generated using OriginPro 2024 (OriginLab Corporation, USA) or GraphPad Prism 8 (GraphPad Software Inc., USA).

## 3 Results

### 3.1 Glucose Availability Enhances Photoheterotrophic Growth but Limits Net CO_2_ Reduction

**FIGURE 1:**
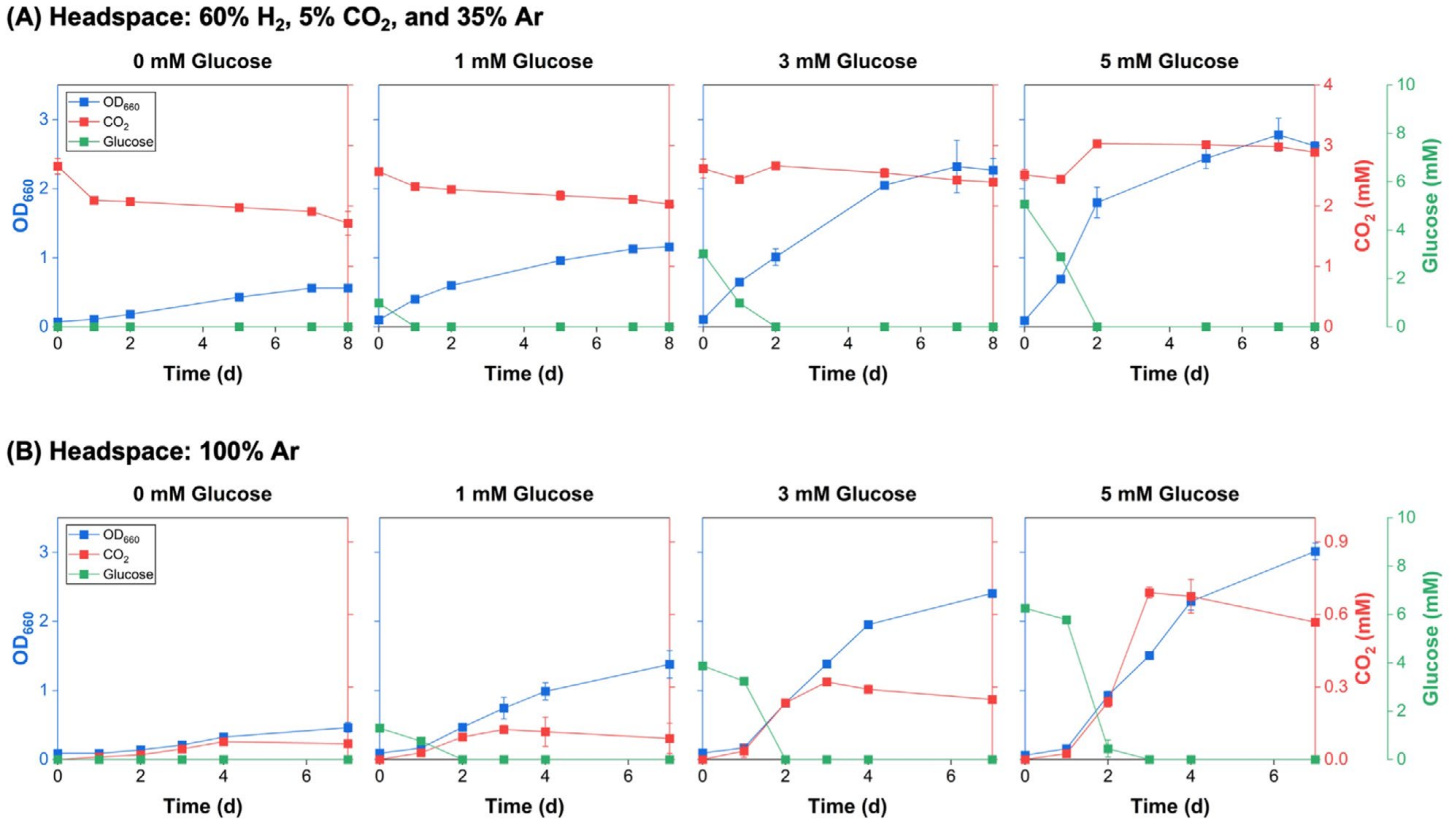
Effect of initial glucose concentrations on growth and CO_2_ dynamics in non‐adapted *Rhodobacter sphaeroides* . Cultures were grown with 0, 1, 3, or 5 mM initial glucose under (A) a gas mixture of 60% H_2_, 5% CO_2_ and 35% Ar, or (B) 100% Ar atmosphere. Cell growth (OD_660_, blue), headspace CO_2_ concentration (red) and residual glucose in the medium (green) were tracked over time.

To establish a metabolic baseline prior to trophic adaptation, *R. sphaeroides* was cultivated under phototrophic conditions with varying initial glucose concentrations (0, 1, 3, or 5 mM) in sealed serum bottles containing a headspace gas mixture of 60% H_2_, 5% CO_2_, and 35% Ar (Figure 1A). This hydrogen‐rich, CO_2_‐containing environment was designed to support potential autotrophic metabolism while allowing assessment of how exogenous organic carbon influences growth and CO_2_ dynamics in non‐adapted cells.

In the absence of glucose, cells grew slowly, reaching an OD_660_ of 0.56 by 8 days. Concurrently, headspace CO_2_ concentration declined from 2.66 to 1.71 mM, a trend that indicates partial CO_2_ depletion likely associated with photoautotrophic activity. This behaviour aligns with the known capacity of *R. sphaeroides* to utilise CO_2_ and H_2_ under light‐driven conditions (Lee et al. 2022). By contrast, glucose supplementation markedly enhanced cell growth. Cultures with 3 and 5 mM glucose reached an OD_660_ of 2.27 and 2.62, respectively, after 8 days of cultivation. However, the difference in biomass between 3 and 5 mM glucose conditions was modest, suggesting growth saturation or diversion of excess carbon into storage metabolites (Touloupakis et al. 2021). Glucose was entirely consumed within the first 2 days in all glucose‐supplemented cultures, indicative of rapid uptake and preferential use of organic carbon sources (Dhar et al. 2023). Headspace CO_2_ dynamics showed a biphasic pattern. For example, in the 5 mM glucose condition, CO_2_ rose from 2.52 mM to a peak of 3.03 mM by 2 days, followed by a gradual decline to 2.89 mM by 8 days. This pattern suggests the initial increase was due to CO_2_ release from glucose catabolism, whereas the subsequent decline reflects partial reassimilation after substrate depletion.

We further evaluated this metabolic response under 100% Ar conditions to determine whether *R. sphaeroides* could re‐assimilate its own metabolically released CO_2_, thereby approaching a net‐zero carbon balance without relying on external CO_2_ uptake (Figure 1B). In the glucose‐supplemented cultures, rapid glucose consumption, enhanced biomass accumulation, and biphasic CO_2_ dynamics were all observed, closely resembling the patterns seen under the H_2_/CO_2_/Ar gas mixture (Figure 1A). However, although CO_2_ reutilization occurred, the extent of reduction was modest. For instance, in the 5 mM glucose condition, CO_2_ concentrations decreased only from a peak of 0.69 mM to 0.57 mM, indicating that complete CO_2_ conversion was not achieved under these conditions. Overall, these findings demonstrate that while *R. sphaeroides* can partially recycle internally produced CO_2_ under mixotrophic conditions, glucose supplementation promotes rapid photoheterotrophic growth at the expense of net CO_2_ assimilation. This outcome underscores the need for metabolic strategies that enhance autotrophic activity in carbon recycling bioprocesses.

### 3.2 Autotrophic Adaptation Enhances CO_2_ Reassimilation Capacity, but Its Effect Diminishes With Sustained Organic Carbon Exposure

Therefore, to address this limitation, we used ALE to enhance the autotrophic capacity of *R. sphaeroides* . We then assessed how this trophic adaptation influences CO_2_ metabolism by comparing heterotrophically adapted (R‐Het) and autotrophically adapted (R‐Auto) populations, both cultured under identical conditions with 1 mM glucose and an Ar headspace.

**FIGURE 2:**
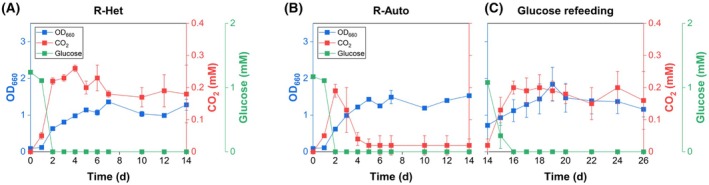
Influence of trophic adaptation on growth and CO_2_ dynamics under 100% Ar atmosphere with 1 mM glucose. Time‐course profiles of growth (OD_660_, blue), headspace CO_2_ (red) and glucose concentration (green) are shown for (A) heterotrophically adapted *R. sphaeroides* (R‐Het), (B) autotrophically adapted cells (R‐Auto) and (C) R‐Auto with a second glucose refeeding at 14 days.

In both groups, glucose was completely consumed within 2 days (Figure 2). This indicates that the adaptation history did not affect the ability to metabolise glucose. However, CO_2_ profiles diverged significantly. In the R‐Het, CO_2_ accumulated gradually, reaching a peak of 0.25 mM at 4 days and slightly decreasing to 0.18 mM by 14 days (Figure 2A). In contrast, CO_2_ in the R‐Auto peaked at 0.19 mM at 2 days and declined sharply to 0.02 mM by 5 days (Figure 2B). This more substantial reduction indicates that CO_2_ generated from glucose catabolism was more effectively reassimilated in R‐Auto, reflecting stronger activation of photoautotrophic metabolism due to prior autotrophic adaptation. Final OD_660_ values also differed: R‐Auto reached 1.53, while R‐Het cultures reached 1.28. This suggests that CO_2_ fixation contributed to enhanced biomass formation in R‐Auto, which retained stronger photoautotrophic activity.

To further evaluate the durability of this phenotype, we re‐supplemented 1 mM glucose to the R‐Auto after the initial 14 days and continued cultivation for an additional 12 days (Figure 2B). During the subsequent 12 days, CO_2_ increased to 0.20 mM at 2 days and declined to 0.16 mM by 12 days. Although some CO_2_ was still reassimilated, the reduction was less pronounced, suggesting that sustained exposure to organic carbon can attenuate autotrophic activity. Overall, autotrophic adaptation enhances CO_2_ reassimilation under photoheterotrophic conditions, but this metabolic advantage diminishes under sustained heterotrophic growth. To overcome this limitation, maintaining efficient CO_2_ reassimilation will likely require strategies that stabilise and balance distinct trophic activities.

### 3.3 A Gas‐Linked Co‐Culture System Promotes Cooperative Carbon Cycling and Enhances Metabolite Productivity

**FIGURE 3:**
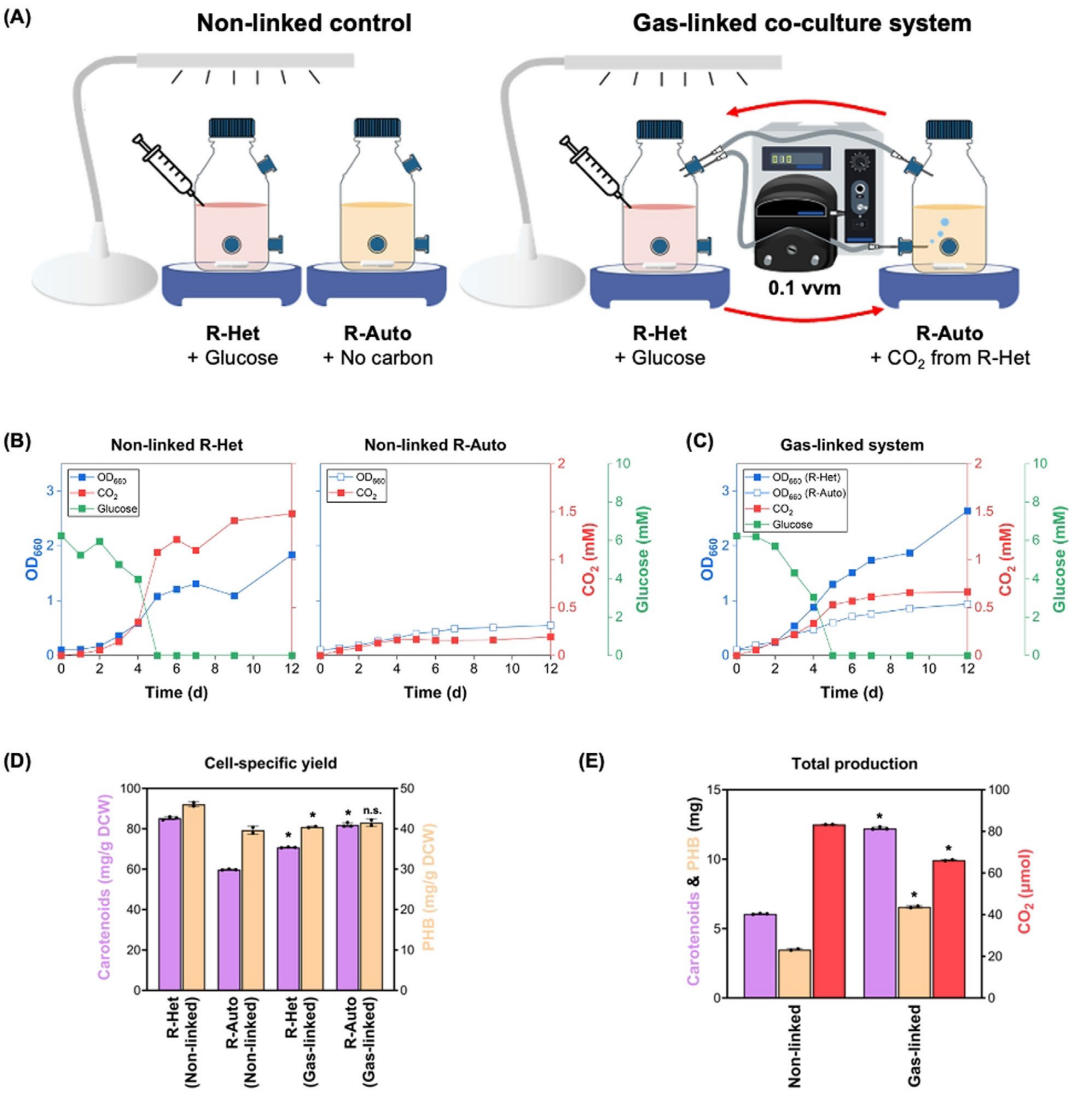
Comparison of non‐linked vs. gas‐linked co‐culture systems under Ar atmosphere. (A) Schematic of the co‐culture setup. R‐Het (5 mM glucose) and R‐Auto (no glucose) were cultured either in physically separated, gas‐isolated chambers (non‐linked) or in chambers sharing a common headspace (gas‐linked). (B) Growth, headspace CO_2_ and glucose profiles for the R‐Het and R‐Auto under the non‐linked system. (C) Growth, CO_2_ and glucose profiles in the gas‐linked system, where gases diffused between R‐Het and R‐Auto chambers. Glucose levels represent the heterotrophic chamber; CO_2_ concentration reflects the shared headspace. (D) Cell‐specific yields of carotenoids (purple bars) and polyhydroxybutyrate (PHB; light orange bars) after 12 days of cultivation. Comparisons were made between the non‐linked and gas‐linked systems for each culture type (R‐Het and R‐Auto). Asterisks indicate significant differences (*p* < 0.05); n.s., not significant. (E) Total carotenoids, PHB and net CO_2_ accumulation after 12 days of cultivation, calculated by summing the values of R‐Het and R‐Auto within each system (non‐linked and gas‐linked). Statistical differences between systems are indicated (*p* < 0.05).

Given the limited CO_2_ reassimilation observed under sustained heterotrophic conditions, we developed a gas‐linked co‐culture system consisting of two physically separated but gas‐connected chambers: one with the R‐Het (with glucose) and the other with the R‐Auto (without glucose). This setup allowed CO_2_ generated by the heterotrophic culture to diffuse into the autotrophic chamber, enabling metabolic coupling via gas‐phase exchange (Figure 3A). For comparison, a control setup was implemented in which each chamber was incubated independently without gas exchange (non‐linked culture).

In the heterotrophic chamber, the gas‐linked configuration supported greater growth (OD_660_ = 2.64) than the non‐linked control (OD_660_ = 1.84) (Figure 3C vs. Figure 3B). This enhancement is likely attributable to the removal of CO_2_, alleviating its potential inhibitory effects on central metabolic pathways such as the TCA cycle. Accordingly, glucose appears to have been preferentially used for biomass formation rather than diverted into secondary metabolites (Montoya‐Vallejo et al. 2023). Consistent with this, carotenoid and PHB yields in the gas‐linked R‐Het were lower (PHB: 40.46 mg/g DCW; carotenoids: 70.80 mg/g DCW) than those in the non‐linked culture (PHB: 46.09 mg/g DCW; carotenoids: 85.28 mg/g DCW) (Figure 3D), suggesting reduced carbon flux into both storage polymers (PHB) and pigments (carotenoids). Similarly, in the autotrophic chamber, gas linkage significantly enhanced growth (OD_660_ = 0.94 vs. 0.55 in the non‐linked culture) (Figure 3C vs. Figure 3B). This indicates that the R‐Auto successfully utilised the CO_2_ diffused from the heterotrophic chamber. In addition, carotenoid yields increased markedly under gas‐linked conditions (non‐linked: 59.80; gas‐linked: 81.96 mg/g DCW) (Figure 3D), consistent with previous reports showing that CO_2_ fixation under light promotes carotenoid biosynthesis closely linked to phototrophic metabolism (Lee et al. 2022).

Importantly, the enhanced growth in both chambers was accompanied by a substantial increase in the total production of value‐added metabolites in the gas‐linked system. PHB and carotenoid production reached 6.56 mg and 12.22 mg, respectively, compared to 3.49 mg and 6.06 mg in the non‐linked control (Figure 3E). At the same time, CO_2_ accumulation in the gas‐linked system was 66.39 μmol, representing a 20.62% reduction relative to 83.60 μmol in the non‐linked control (Figure 3E). These findings demonstrate that the gas‐linked co‐culture system not only promotes cooperative carbon cycling and thereby reduces CO_2_ emissions but also increases the production of value‐added metabolites.

## 4 Discussion

### 4.1 *Rhodobacter sphaeroides* as a Model Bacterium

*Rhodobacter sphaeroides* was selected as the model system in this study due to its remarkable metabolic flexibility, which enables growth under photoheterotrophic, photoautotrophic, and chemoheterotrophic conditions (Yu et al. 2022). This versatility stems from its modular energy‐generation systems and dynamic regulation of carbon assimilation pathways in response to environmental cues such as light intensity, redox status and substrate availability. Under photoheterotrophic conditions, *R. sphaeroides* utilises light as an energy source while metabolising organic carbon sources. The highly efficient light‐harvesting complexes and branched electron transport chain in the bacterium enable ATP synthesis via cyclic photophosphorylation, independently of external electron acceptors (Li et al. 2021). In photoautotrophic mode, *R. sphaeroides* fixes CO_2_ using electrons derived from an inorganic donor, with the CBB cycle serving as the primary pathway for converting CO_2_ into cellular biomass (Gibson et al. 2002). In the absence of light, it can switch to chemoheterotrophic respiration utilising oxygen or alternative terminal electron acceptors (Pappas et al. 2004).

Importantly, *R. sphaeroides* also synthesises high‐value intracellular metabolites across trophic modes. Under carbon‐rich, nitrogen‐limited nutrient imbalance, it accumulates PHB—a biodegradable bioplastic that serves as a carbon‐storage polymer—by diverting excess carbon flux through the acetyl‐CoA node into PHB biosynthesis (Lee, Fitriana, et al. 2020). During phototrophic growth, it produces carotenoid pigments that protect the photosynthetic apparatus by mitigating photo‐oxidative stress (Lee et al. 2022; Yu, Park, et al. 2023)—compounds with broad industrial relevance as natural colorants, antioxidants and nutraceutical ingredients (Shanaida et al. 2025). Additionally, *R. sphaeroides* is capable of biohydrogen production via nitrogenase activity under anaerobic phototrophic conditions, making it a candidate for sustainable biohydrogen production (Li et al. 2021).

Compared to obligate photoautotrophs such as algae or cyanobacteria, *R. sphaeroides* exhibits faster growth and higher metabolite yields across trophic regimes (Orsi et al. 2021). Combined with its genetic tractability and well‐characterised physiology, this makes it an ideal chassis for investigating carbon flux and bioproduct synthesis in synthetic microbial systems. In this study, *R. sphaeroides* was employed in both heterotrophic and autotrophic configurations, achieved through prior trophic adaptation. This approach allowed for controlled analysis of carbon exchange and metabolic interactions within a single species, avoiding the complexity and variability inherent to multispecies consortia.

### 4.2 Physiological Adaptation Strategies in Biorefinery Applications

Microbial biorefineries commonly utilise heterotrophic metabolism for high‐yield production of metabolites; however, this mode inherently releases CO_2_ as a byproduct (François et al. 2020). Recent efforts have aimed to integrate autotrophic and heterotrophic pathways to recycle CO_2_ emissions while maintaining product output (Yu, Shin, et al. 2023). Strategies include engineering of heterotrophs with photosynthetic CO_2_‐fixation pathways (e.g., introducing the CBB cycle into *Escherichia coli* ) (Lee, Kim, et al. 2020) and expressing heterotrophic biosynthetic pathways in autotrophs (e.g., 1‐butanol production in *Synechocystis*) (Liu et al. 2019). However, these approaches often face limitations due to regulatory hurdles, genetic instability and metabolic burden associated with heterologous gene expression (Eckerstorfer et al. 2025; Francis and Page 2010; Puiggené et al. 2025).

As a non‐genetically modified organism (GMO) alternative, ALE offers a powerful tool for enhancing microbial traits through selective pressure without introducing foreign DNA (Lee and Kim 2020). ALE involves serial culturing under defined conditions, allowing beneficial mutations to accumulate over time. Although ALE lacks the precision of targeted gene editing, it offers advantages such as regulatory simplicity, lower cost and greater stability—attributes desirable for industrial applications. ALE‐derived strains are generally exempt from transgenic regulatory approval, and the method does not require additional genetic constructs or specialised expression systems (Lee and Kim 2020). These features can lower implementation costs and enhance process flexibility. In the context of CO_2_ metabolism, ALE has been applied to improve traits such as CO_2_ fixation efficiency in *Sporomusa ovata* (Zhang and Tremblay 2018) and autotrophic growth in *Clostridium autoethanogenum* (Heffernan et al. 2024). However, ALE has been rarely applied to *R. sphaeroides* , with a notable example being the study by Atay et al. (2024), which improved cobalt resistance in this species. To our knowledge, no prior studies have used ALE to enhance autotrophic metabolism in *R. sphaeroides* , underscoring the novelty of our approach.

Our findings demonstrate that autotrophic adaptation in *R. sphaeroides* significantly enhances the capacity to reassimilate CO_2_ during photoheterotrophic growth, resulting in an 88.89% reduction in net CO_2_ accumulation (Figure 2). However, this phenotype proved reversible: sustained exposure to glucose diminished CO_2_ fixation capacity. This observation highlights the dynamic nature of physiological adaptation and suggests that, under heterotrophic conditions, maintaining autotrophic activity may require continuous selective pressure or environmental reinforcement. While this study did not directly pursue strategies to reinforce adaptation, further investigations involving genomic and transcriptomic profiling may help elucidate the molecular basis of such metabolic plasticity and guide strategies to stabilise ALE‐derived traits (Lee and Kim 2020).

### 4.3 Implications and Limitations of Gas‐Linked Co‐Culture Systems

Microbial co‐culture broadly refers to the simultaneous cultivation of two or more microbial species within the same system, where they interact either directly through cell–cell contact or indirectly through the exchange of metabolites (Ravikrishnan et al. 2020). The most basic configuration is mixed co‐culture, in which all strains are inoculated into a shared medium. While operationally simple, this approach often suffers from limitations such as uncontrolled population dynamics, metabolic competition, and unintended growth inhibition, especially when the strains differ in optimal growth conditions or metabolic requirements (Song et al. 2024). To address these limitations, physically compartmentalised co‐culture systems have been developed. These include membrane‐based systems that allow selective diffusion of metabolites, and immobilised systems where cells are confined within matrices like agar (Song et al. 2024). However, most of these systems focus on the exchange of liquid‐phase metabolites (e.g., organic acids, sugars), with few addressing interactions mediated by gaseous intermediates. In the context of integrating autotrophic and heterotrophic metabolisms, establishing systems that share only the gaseous phase—rather than direct contact or compartmentalization within the same liquid medium—can offer a more efficient and controllable means of metabolic interaction. One example of gas‐mediated integration is the gas separation membrane bioreactor (GS‐MBR), in which gases produced during fermentation (e.g., CO_2_ and H_2_) are selectively separated and repurposed for downstream biological reactions (Bakonyi et al. 2018). For instance, CO_2_ can be routed to phototrophic or autotrophic organisms for assimilation, while H_2_ is recovered as biohydrogen. Although promising, GS‐MBR systems often require complex infrastructure and precise pressure control, limiting their accessibility and scalability (Bakonyi et al. 2018).

In this study, we present a conceptually simpler yet functionally effective gas‐linked co‐culture system. By maintaining heterotrophically and autotrophically adapted *R. sphaeroides* populations in physically separated chambers linked via a shared headspace, we enabled continuous gas‐phase exchange without any mixing of the liquid phases. This design allowed each population to grow under its respective adapted conditions while engaging in functional metabolic coupling. Compared to non‐linked controls, the gas‐linked system yielded multiple benefits (Figure 3). First, the heterotrophic chamber showed enhanced growth, likely due to alleviation of CO_2_ inhibition in central metabolism. Second, the autotrophic chamber exhibited significant biomass accumulation and increased carotenoid yield, indicating successful utilisation of heterotroph‐generated CO_2_. Most importantly, system‐wide metabolite production (carotenoids and PHB) was substantially higher (2.02‐fold and 1.88‐fold, respectively), and net CO_2_ accumulation was reduced by 20.62%, which confirms effective intra‐system carbon recycling.

The spatial separation inherent to gas‐linked designs allows each population or species to operate within its own optimised medium, mitigating competition and compatibility issues that often hinder traditional mixed co‐cultures. This configuration also enables distinct environmental settings, such as differential light exposure or temperature control, thereby expanding the versatility of such systems. Although this study employed a single bacterial species differentiated only by trophic adaptation history, the underlying framework could be extended to interspecies co‐cultures involving autotrophic and heterotrophic strains, providing a foundation for more complex microbial consortia. However, while our initial goal was to establish a net‐zero system for value‐added metabolite production, complete CO_2_ elimination was not achieved. This outcome highlights a key limitation of the current design and the need for further optimisation to fully realise the potential of gas‐linked co‐culture systems. Potential avenues for improvement include tuning the ratio of heterotrophic to autotrophic chamber volumes, optimising light intensity and nutrient formulations, and implementing continuous electron donor supplementation to sustain autotrophic activity. Collectively, although further improvements are required, we believe that this study provides a new perspective on the integration of heterotrophic and autotrophic metabolisms through gas‐phase linkage.

## 5 Conclusions

This study presents a gas‐linked co‐culture system that enables spatially separated yet metabolically connected microbial interactions, aiming to enhance carbon efficiency and sustainable production of value‐added metabolites. By utilising heterotrophically and autotrophically adapted *R. sphaeroides* strains in physically separated chambers, we enabled dynamic CO_2_ exchange between compartments. The configuration resulted in improved biomass accumulation, increased total production of carotenoids and PHB, and a measurable reduction in net CO_2_ emissions. These findings demonstrate that gas‐linked microbial co‐cultures represent a viable platform for coupling carbon fixation with bioproduct synthesis. While further optimisation is required to achieve a fully net‐zero process, this modular approach is expected to provide a promising foundation for sustainable biorefinery development.

## Author Contributions

**Jaeyoung Yu:** conceptualisation, data curation, writing – original draft. **Danbee Kim:** conceptualisation, investigation, writing – review and editing. **Jiye Lee:** investigation, methodology, writing – review and editing. **Hui Su Kim:** formal analysis. **Hwi Jong Jung:** visualization. **Yuri Kim:** writing – review and editing. **Sahng Hyuck Woo:** project administration. **Eunsung Kan:** funding acquisition. **Jeong Hyeon Kim:** validation. **Soo Youn Lee:** conceptualisation, funding acquisition, supervision, writing – original draft.

## Funding

This work was supported by the National Research Foundation (NRF) of Korea, RS‐2022NR067354, RS‐2024‐00337717.

## Conflicts of Interest

The authors declare no conflicts of interest.

## Data Availability

The data that support the findings of this study are available from the corresponding author upon reasonable request.
